# A Patient Stratification Approach to Identifying the Likelihood of Continued Chronic Depression and Relapse Following Treatment for Depression

**DOI:** 10.3390/jpm11121295

**Published:** 2021-12-04

**Authors:** Rob Saunders, Zachary D. Cohen, Gareth Ambler, Robert J. DeRubeis, Nicola Wiles, David Kessler, Simon Gilbody, Steve D. Hollon, Tony Kendrick, Ed Watkins, David Richards, Sally Brabyn, Elizabeth Littlewood, Debbie Sharp, Glyn Lewis, Steve Pilling, Joshua E. J. Buckman

**Affiliations:** 1 Centre for Outcomes Research and Effectiveness (CORE), Research Department of Clinical, Educational & Health Psychology, University College London, 1-19 Torrington Place, London WC1E 7HB, UK; r.saunders@ucl.ac.uk (R.S.); s.pilling@ucl.ac.uk (S.P.); 2 Department of Psychiatry, University of California, Los Angeles, CA 90095, USA; zachary.d.cohen@gmail.com; 3 Statistical Science, University College London, 1-19 Torrington Place, London WC1E 7HB, UK; g.ambler@ucl.ac.uk; 4 Department of Psychology, School of Arts and Sciences, 425 S. University Avenue, Philadelphia, PA 19104, USA; derubeis@psych.upenn.edu; 5 Centre for Academic Mental Health, Population Health Sciences, Bristol Medical School, University of Bristol, Oakfield House, Bristol BS8 2BN, UK; nicola.wiles@bristol.ac.uk; 6 Centre for Academic Primary Care, Population Health Sciences, Bristol Medical School, University of Bristol, Canynge Hall, Bristol BS8 2PS, UK; David.Kessler@bristol.ac.uk (D.K.); Debbie.Sharp@bristol.ac.uk (D.S.); 7 Department of Health Sciences, University of York, ARRC Building, Heslington, York YO10 5DD, UK; simon.gilbody@york.ac.uk (S.G.); sally.brabyn@york.ac.uk (S.B.); liz.littlewood@york.ac.uk (E.L.); 8 Department of Psychology, Vanderbilt University, Nashville, TN 37235, USA; steven.d.hollon@vanderbilt.edu; 9 Primary Care Research Centre, Primary Care, Population Sciences and Medical Education, Faculty of Medicine, University of Southampton, Aldermoor Health Centre, Southampton SO16 5ST, UK; A.R.Kendrick@soton.ac.uk; 10 Department of Psychology, University of Exeter, Sir Henry Wellcome Building for Mood Disorders Research, Perry Road, Exeter EX4 4QG, UK; E.R.Watkins@exeter.ac.uk; 11 Institute of Health Research, University of Exeter College of Medicine and Health, Exeter EX1 2LU, UK; D.A.Richards@exeter.ac.uk; 12 Department of Health and Caring Sciences, Western Norway University of Applied Sciences, Inndalsveien 28, 5063 Bergen, Norway; 13 Division of Psychiatry, University College London, Maple House, London W1T 7NF, UK; glyn.lewis@ucl.ac.uk; 14 Camden & Islington NHS Foundation Trust, St. Pancras Hospital, 4 St. Pancras Way, London NW1 0PE, UK; 15 iCope—Camden & Islington Psychological Therapies Services—Camden & Islington NHS Foundation Trust, St. Pancras Hospital, 4 St. Pancras Way, London NW1 0PE, UK

**Keywords:** depression, primary care, latent profile analysis, personalised medicine, patient stratification

## Abstract

Background: Subgrouping methods have the potential to support treatment decision making for patients with depression. Such approaches have not been used to study the continued course of depression or likelihood of relapse following treatment. Method: Data from individual participants of seven randomised controlled trials were analysed. Latent profile analysis was used to identify subgroups based on baseline characteristics. Associations between profiles and odds of both continued chronic depression and relapse up to one year post-treatment were explored. Differences in outcomes were investigated within profiles for those treated with antidepressants, psychological therapy, and usual care. Results: Seven profiles were identified; profiles with higher symptom severity and long durations of both anxiety and depression at baseline were at higher risk of relapse and of chronic depression. Members of profile five (likely long durations of depression and anxiety, moderately-severe symptoms, and past antidepressant use) appeared to have better outcomes with psychological therapies: antidepressants vs. psychological therapies (OR (95% CI) for relapse = 2.92 (1.24–6.87), chronic course = 2.27 (1.27–4.06)) and usual care vs. psychological therapies (relapse = 2.51 (1.16–5.40), chronic course = 1.98 (1.16–3.37)). Conclusions: Profiles at greater risk of poor outcomes could benefit from more intensive treatment and frequent monitoring. Patients in profile five may benefit more from psychological therapies than other treatments.

## 1. Introduction

Depression is amongst the most burdensome diseases across the globe [1]. It is highly prevalent, affecting approximately 320 million people annually [2,3], results in significant impairment for prolonged periods of time [4], and is typically thought to follow a ‘relapsing-remitting’ course, with multiple episodes throughout life [5]. Much of our understanding regarding the course of depression comes from general population studies of adults [6,7], but clinical samples are needed to ensure greater relevance to healthcare settings. This is especially important as the course of depression is quite different for those that seek treatment compared to those that do not, with the rate of relapse much higher in clinical samples [8,9].

Treatments for depression including continued use of antidepressant medications can be effective in reducing the risk of relapse and of chronic depression [10]. Although many patients do not want long-term treatment with antidepressants [11], there is an increasing trend for them to be maintained on antidepressants for two years or more [12,13], and the use of antidepressants has greatly increased over the last three decades [14]. Although pharmacogenetics may hold promise for personalizing antidepressant treatments [15,16], it is becoming increasingly common that antidepressants are prescribed indefinitely for all patients with a history of previous relapses, to mitigate the risks of chronic illness and relapse [11,12,17,18]. This is despite evidence of the waning effectiveness of medications over time [19,20,21,22], and even increased risk of relapse and other harmful outcomes for those remaining on medications after several years [17,23]. Some psychological therapies offer protection against relapse which is equivalent to that offered by continuation of antidepressants at six months post-treatment [24,25,26]. Psychological therapies can also be used after the acute phase of treatment, i.e., when a patient reaches remission, these can have a preventive effect [27,28], reducing the risk of relapse by approximately 15–29% up to two years post-treatment [29]. Further, combining antidepressants and psychotherapies or augmenting one with the other when remission has not been achieved with monotherapy improves the chances of avoiding relapse [27,30,31]. However, the benefits of such treatments are not universal, the costs of either treatment can be prohibitive, and psychological treatments in particular are not readily available throughout the world [29,32]. Coupled with the prevalence of depression, this makes it impractical to offer prophylactic treatment to all patients with depression. As such, identifying subgroups of patients for whom further treatment might reduce the likelihood of continued chronic depression or the risk of relapse could have important implications in terms of clinical outcomes and reduced healthcare costs.

Identifying subgroups of patients by better understanding the complex relationships between pre-treatment patient characteristics can allow for the development of ‘clinical phenotypes’ [33], informing prognosis and supporting treatment decision making. One way of doing so is via latent profile analysis (LPA), which is part of the latent variable mixture modelling family of analytic approaches. Based on probability theory, these models identify homogeneous subpopulations by identifying stratified groups of patients [34,35] based on the relationships between available indicator variables (both continuous and categorical variables can be used in the LPA). These methods have been applied to a range of clinical presentations, including in Alzheimer’s disease [36], Crohn’s disease [37], and depression and anxiety [38], where they have also been used to predict likely treatment outcomes for people receiving psychological therapy in routine care settings [39,40]. However, such methods have not been applied to consider the longer-term prognosis of patients after the end of acute-phase treatment for depression, including continued chronic depression or relapse. A number of studies have attempted to develop models to predict relapses to depression [41,42,43]. However, these have been hampered by methodological problems, including small sample sizes, inappropriate handling of missing data, and a lack of validation, and they have largely failed to make accurate predictions [44]. Other studies to develop more accurate models are planned [45], but no findings are available yet, and the clinical utility of any such models are yet to be established. Patient stratification techniques such as LPA offer an alternative means of predicting outcomes which may have greater face-validity for clinicians and patients because the characteristics of each profile can be described in more accessible terms [40]. This is often not possible with regression based or supervised machine learning models because the reasons for any individual being classified as having a particular degree of risk for the outcome of interest may be hard to determine and often harder still to explain when there are multiple predictor variables [46,47]. Furthermore, variable-centred approaches such as regression modelling require a different model, and therefore a different set of associations, by outcomes, whereas patient stratification approaches, following the identification of profiles, can allow exploration of associations between profiles and multiple different outcomes [48]. The application of latent profiling methods to stratify patients and identify individual risks of poor longer-term prognosis might therefore have important clinical utility.

The aims of this study were to identify the stratified profiles of adults treated for depression in primary care settings, based on pre-treatment characteristics, and explore differences in the course of illness and risk of relapse between patients in these profiles. The associations between the types of treatment received on outcomes within each profile were also explored.

## 2. Materials and Methods

A pre-registered protocol for this study and analytic plan is available through the Open Science Framework (DOI 10.17605/OSF.IO/Y52HG) (https://osf.io/y52hg/ accessed on 29 July 2021).

### 2.1. Participants

The Depression in General Practice (Dep-GP) individual patient dataset [49] was used to derive the sample for the current analysis. The overall aims of the Dep-GP project are to identify prognostic factors associated with outcomes from treatment for depression in primary care. The dataset contains individual patient data from the participants of 12 randomised controlled trials (RCTs) of treatments for depression. Information regarding the dataset and protocol for the methods of identifying relevant studies for the Dep-GP dataset are available elsewhere [49,50]. Importantly, all trials included in the Dep-GP dataset were of adults with depression who had been recruited from general practices and used the most common comprehensive diagnostic and screening measure of depression and anxiety disorders used in primary care RCTs—the Revised Clinical Interview Schedule (CIS-R) [51].

#### Inclusion Criteria

For this analysis, individual patient data were sought from RCTs of adults with depression recruited in primary care included in the Dep-GP database, that: used the CIS-R at baseline, had an endpoint at 3 to 4 months post-baseline, and collected data on depressive symptoms at follow-up periods after the 3 to 4 month post-baseline endpoint. Seven studies in the Dep-GP database met these criteria (presented in Table 1).

### 2.2. Measures

The relevant measures in the seven included studies were:

CIS-R [51]: establishes the nature and severity of common mental disorder symptoms and provides primary and secondary diagnoses according to ICD-10 criteria. CIS-R is made up of 14 symptom subsections. Five subscales contribute to a depression score, covering core features of depression, depressive thoughts, fatigue, concentration and forgetfulness, and sleep; the remaining subscales cover anxiety disorders and related symptoms (generalized anxiety, worry, irritability, somatic anxiety/hypochondriasis, panic, obsessions, compulsions, phobias (split into agoraphobia, social phobia, and specific phobias). Each section includes information on the duration of the problems assessed, and there is an additional subsection that covers general health and disabilities, none of which contributes to either the depression or anxiety total scores. The total scores are calculated by summing the scores of the contributing individual subscales.

Beck Depression Inventory (BDI-II) [52]: This 21-item assessment is used to measure depressive symptoms; each item is scored 0–3 with a maximum score obtainable of 63. A cut-off of ≥14 is used to indicate significant symptoms of depression and ≤10 is used for remission.

Patient Health Questionnaire (PHQ-9) [53]: This is a 9-item depression screening measure. Items are scored 0–3; a cut-off of ≥10 is used to indicate “caseness”, that is symptoms of depression likely to be commensurate with reaching diagnostic criteria for a major depressive episode. A cut-off of ≤9 is used for remission.

Edinburgh Postnatal Depression Scale (EPDS) [54]. This 10-item measure assesses symptoms of depression among women in the post-natal period. Each item is scored 0–3 and the maximum obtainable score is 30, with scores of ≥13 indicative of caseness. A cut-off of ≤12 is used for remission.

In addition, socio-demographics including age, gender, ethnicity, and employment status were also available.

### 2.3. Outcomes

Two primary outcomes were included in this analysis, with participants included in the analyses of one outcome only, depending on whether they were or were not in remission at 3 to 4 months:

(1) Relapse: This outcome is for patients who were in remission at 3 to 4 months and is defined as not in remission at either 6 to 8 or 9 to 12 months on the primary depressive symptom measure used (either BDI-II, PHQ-9, or EPDS) (See Table 1 for primary outcomes).
(2) Continued chronic course of depression: This outcome is for patients not in remission at 3 to 4 months and is defined as caseness at all subsequent time-points on the primary symptom measure used in the study.

### 2.4. Data Analysis

#### 2.4.1. Latent Profile Analysis (LPA)

The selection of variables to include in the profiling (termed indicator variables, which can be continuous or categorical) was determined from previous analyses, identifying prognostic factors in the dataset and findings from LPA in similar settings. Baseline depression severity, as well as the duration of depressive illness, have both been identified as strong prognostic factors of treatment outcome, as have initial anxiety symptom severity levels and the duration of comorbid anxiety disorders [50]. In addition to these, the age of participant as well as the employment status of participant were considered as indicators, given their role in discriminating profiles in previous LPA [39]. Seven patient factors, identified at baseline, were included in the LPA. These factors, and how they were transformed for the current analysis, are listed below:

(a) The sum of the scores on the depression sub-scales of the CIS-R;
(b) The sum score of the anxiety subscales of the CIS-R:
(c) Duration of depression made binary: less than one year, or one year, or greater;
(d) Duration of non-depressive (i.e., anxiety) symptoms measured on the CIS-R made binary: less than one year, or one year or greater;
(e) History of antidepressant use (yes/no);
(f) Age at baseline;
(g) Employment status (employed or unemployed).

A number of metrics were used to identify the optimal LPA solution, in line with established guidance and previous studies employing this analytic approach [39,55]. The following metrics were considered together, with the decision regarding the optimal solution based on a balance between evidence provided by the Vuong-Lo-Mendell-Rubin Likelihood Ratio test (VLMR-LRT) [56], the Bayesian Information Criteria (BIC), sample-size adjusted BIC (SABIC), and entropy values of the model. The VLMR-LRT tests the K model (specified model with K profiles) against the model with one less profile (K-1 model), and a *p*-value less than 0.05 is taken to indicate the K model is a better fit to the data (a *p*-value >0.05 indicates little evidence that the K model provides a better fit than the K-1 model). Lower BIC and SABIC values are considered to indicate better model fit, and higher entropy values indicate better classification accuracy of the model. LPA was conducted in Mplus V8 [57].

##### Association between Profiles and Outcomes

Once the best fitting profile solution was identified, all individuals were allocated to the profiles to which they had the highest probability of membership. Logistic regression models were constructed for both primary outcomes, with profile included as an independent categorical variable to explore differences in outcomes between profiles. All regression analyses were performed in Stata16 [58]; odds ratios (OR) and 95% confidence intervals (95% CI) are presented.

First, differences in the likelihood of relapse or of continued chronic course of depression between the profiles were explored by constructing logistic regression models with allocated treatment (within the RCT, 15 levels in total) included as a covariate (variables used in profiling were not included as covariates in regression models). The difference in each outcome across the profiles as a whole was initially explored through an omnibus test of the variance explained by the model (i.e., an F-test). Because there was evidence of such a difference, the above analyses were conducted comparing the outcomes for participants in each profile relative to both the largest profile as well as another profile which had the average likelihood of remission at 3 to 4 months. The adjusted probability of each outcome was presented for each identified profile, with forest plots constructed to display these differences.

Second, the likelihood of relapse or continued chronic course of depression was compared when participants were randomised to (1) psychological treatments, (2) antidepressant treatment, or (3) ‘Treatment as usual’ or usual care (TAU) arms in their study. Two of the interventions listed in Table 1 (Listening intervention and Physical activity), made up a fourth group labelled ‘Other’, but this was not used in this set of analyses owing to very small numbers of available participants. These groupings were chosen to maximise power whilst providing an informative comparison between intervention types. An omnibus test for the difference in each outcome across all profiles whilst fitting an interaction between profile and treatment type was initially conducted. As there was evidence of differences, analyses were conducted within profiles, to estimate differences in the likelihood of each outcome when different treatments were received for members of each profile, in line with previous analyses looking at treatment differences between identified profiles [40].

#### 2.4.2. Missing Data

Missing data on latent profile indicators was handled in Mplus using Full Information Maximum-Likelihood (FIML), following previous analyses using these approaches [39]. For the analysis exploring the association between outcomes and the identified profiles, individuals with missing outcome data (i.e., lost to follow up) had their outcome data imputed; imputation was also conducted for any participants missing baseline data. Missing baseline and outcome data were imputed using multiple imputation with chained equations (MICE) in Stata 16.0 [58]. This approach uses regression models to impute missing values. A number of imputed datasets (in this study we used 50) were produced to reflect the uncertainty/variability in the imputation process. Where data were not reasonably able to be log transformed to meet normality assumptions, predictive mean matching (PMM) via a k-nearest neighbours approach was used [59]; in this study, we used k = 10 nearest neighbours. Linear regression was used for approximately normally distributed continuous variables, logistic regression models for binary variables, and ordinal and multinomial regression models for ordered and unordered categorical variables, respectively. All imputation models were built using data on baseline and outcome variables following conventions [60]. Only variables with less than 50% missing data were imputed (although in practice no variables had more than 50% missing data; see extended data for degrees of missing by variable [49]), and only participants with missing data on fewer than 50% of the variables were included (this resulted in two participants being removed from the IPCRESS study). Sensitivity analyses including only individuals with complete data were performed to check for differences between imputed and observed model results.

### 2.5. Ethical Considerations and Trial Registrations

All included studies were granted ethical approvals and all participants gave informed consent (see Supplementary Materials D for details). No additional NHS ethical approval was required for this study: HRA reference 712/86/32/81.

## 3. Results

### 3.1. Descriptive Statistics

The included sample is presented in Table 2. The mean age was 43 years (sd = 13.8), with 73% of participants identifying as female and nearly 50% in employment. Just under 50% of the sample reported experiencing depression for over 1 year at baseline, and 73% reported significant anxiety symptoms for over a year at baseline.

### 3.2. Latent Profile Analysis

The model fit statistics for the profile-solutions are presented in Supplementary Online Table S1. Overall, considering the model fit and classification accuracy statistics, the seven-profile model solution was selected. There was little evidence that an eight-profile solution provided a better fit to the data than the seven-profile model (VLMR-LRT *p*-value = 0.434), whereas there was evidence that the seven-profile solution was a better fit than the six-profile model (seven-profile VLMR-LRT *p*-value = 0.019). The AIC and BIC values continued decreasing with the increase in the number of profiles, and the entropy value was highest for the seven-profile model, providing further support that the seven-profile model was the best fit for the data. Each participant was allocated to the profile to which they had the highest probability of membership [40]. The characteristics of individual profiles identified are described below and presented in Table 3.

Profile 1: This was the smallest profile identified (making up 5% of the sample) and was characterised by lower baseline depression and anxiety severity scores compared to all other profiles, whilst having a high likelihood of a duration of anxiety disorders over one year long at baseline.

Profile 2: This profile was the youngest group identified (mean = 29.43 years) and was characterised by patients that were very likely to have had depression for less than one year at baseline and were very likely to be unemployed.

Profile 3: Patients in this profile were similar to Profile 2, in that they had severity scores below the study average and were likely to have suffered from depression for less than a year. However, they were older than Profile 2 members on average (mean age = 40) and were very likely to be employed and have a history of antidepressant use.

Profile 4: This was one of the largest profiles (19% of the sample) and had the second highest initial symptom severity scores (after Profile 7), although the duration of depression for patients in this profile was likely to be less than one year long at baseline.

Profile 5: Members of this profile appeared to be more chronic sufferers of depression as they were highly likely to have been suffering from both depression and anxiety for more than one year, and over three quarters had a history of antidepressant use.

Profile 6: This profile had the oldest average age (62 years), were very likely to be unemployed, and very likely to have both a history of using antidepressants and having suffered from anxiety for over a year.

Profile 7: This group presented with the highest average depression and anxiety scores and a very high likelihood of suffering from depression and anxiety for over a year and having a history of antidepressant use. This was the largest profile identified (22% of the sample).

### 3.3. Associations between Profiles and Outcomes

**Figure 1:**
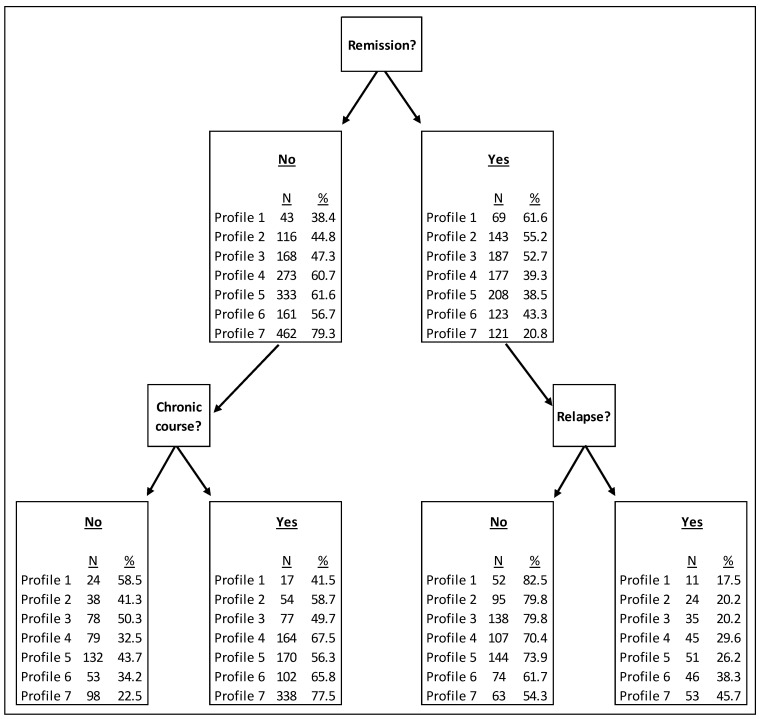
Outcomes between profiles.

Following the identification of the profiles, differences in outcomes between the profiles were explored. Participants were split into those who were in remission at the 3 to 4 months (study) endpoint and were included in the ‘relapse’ outcome analysis, or not in remission and therefore were included in the analysis of the continued chronic course outcome. These categories were mutually exclusive, so participants could not be included in both. Figure 1 presents the proportion of individuals in remission at 3 to 4 months, those who relapsed, and those who followed a continued chronic course of depression, by profile. Profile 1 (low severity) were the most likely to be in remission, not relapse, and not follow a continued chronic course, whereas Profile 7 were least likely to be in remission. Profile 3 (likely first episode, employed) had a near 50% likelihood of remission, a 20% chance of relapse for those previously in remission, and a 50% chance of continued chronic course (if not in remission at 3 to 4 months).

**Figure 2:**
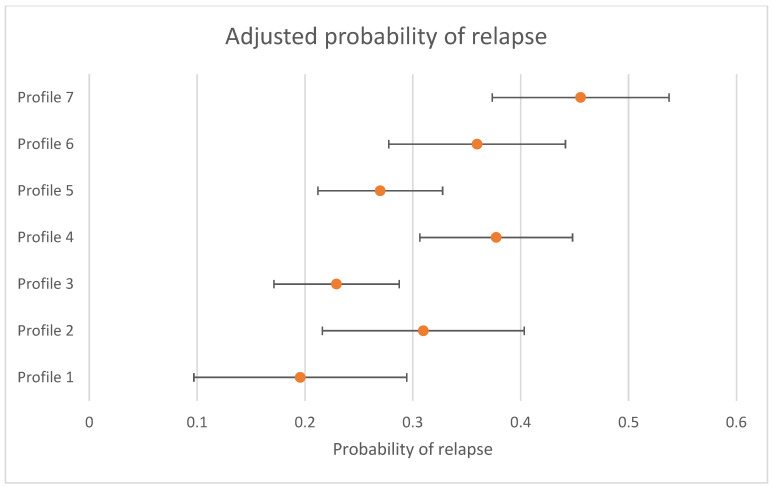
Adjusted probability of relapse by profile (probability and 95% CIs presented).

The differences in the likelihood of relapse for individuals who were in remission at 3 to 4 months between profiles is presented in Table 4. There was evidence that the likelihood of relapse was not the same across all profiles (omnibus test *p* < 0.001). When using Profile 7 (the largest profile) (“high severity”) as the reference group (left side of table), Profile 1 (OR = 0.30 (95% CI = 0.13; 0.68)), Profile 2 (OR = 0.51 (95% CI = 0.27; 0.97)), Profile 3 (OR = 0.38 (95% CI = 0.22; 0.65)), and Profile 5 (OR = 0.44 (95% CI = 0.27; 0.73)) were less likely to relapse. When using Profile 3 (the profile with a 50% likelihood of remission at 3 to 4 months) (“first episode, employed”) as the reference group (right side of Table 3), Profile 4 (OR = 1.82 (95% CI = 1.11; 2.99)), Profile 6 (OR = 1.78 (95% CI = 1.04; 3.04)), and Profile 7 (OR = 2.63 (95% CI = 1.53; 5.43)) were more likely to relapse. The probability of relapse, adjusted by treatment received, and 95% CIs are presented in Figure 2. Sensitivity analyses only conducted with individuals who had complete data at the follow up time periods are presented in Supplementary Online Table S2 and show that all findings were replicated, apart from the decreased odds of relapse for Profile 2 when compared to Profile 7.

**Figure 3:**
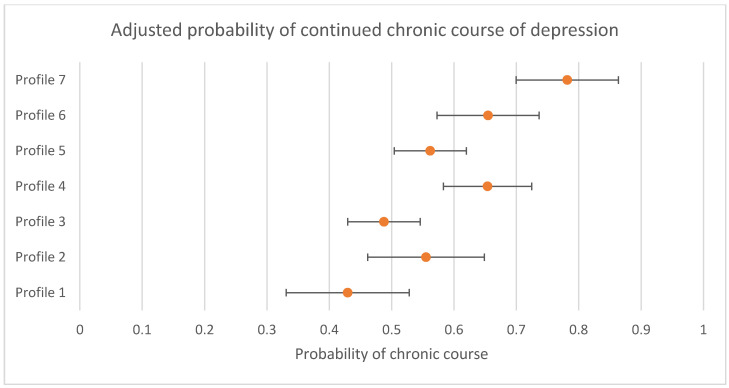
Adjusted probability of continued chronic course of depression by profile (probability and 95% CIs presented).

Associations between profile and differences in the likelihood of continued chronic course of depression for participants not in remission (i.e., still scoring in the clinical range) at 3 to 4 months between profiles are presented in Table 5. There was evidence that the likelihood of a continued chronic course was not the same across all profiles (omnibus test *p* < 0.001). Compared to Profile 7, all other profiles were less likely to follow a continued chronic course, and when compared to Profile 3, Profile 4 (OR = 1.84 (95% CI = 1.24; 2.72)), Profile 6 (OR = 1.77 (95% CI = 1.11; 2.82)), and Profile 7 (OR = 3.15 (95% CI = 2.11; 4.71)), were more likely to follow a continued chronic course. The probability of a continued chronic course with 95% CIs are presented in Figure 3. Sensitivity analyses conducted only with individuals that had complete data at the follow up time periods replicated these findings (presented in Supplementary Online Table S3).

### 3.4. Association between Treatment Type Received and Outcome by Profile

Finally, differences in the likelihood of relapse and of a continued chronic course were compared within each profile when individuals received different types of treatment. We compared the odds of relapse and continued chronic course of depression between individuals who were randomised to: (1) psychological treatments, (2) antidepressant medication(s), or (3) Treatment as usual.

Differences in the odds of relapse between randomised treatments are presented in Table 6. There was evidence that differences between the profiles for each treatment type were not the same (omnibus test *p* < 0.001). There was little evidence of differences in the likelihood of later relapse between treatment types for individuals from any profile, except for Profile 5, where relapse was more likely for individuals who received treatment as usual (OR = 2.51 (95% CI = 1.16; 5.40)) or antidepressants (OR = 2.92 (95% CI = 1.24; 6.87)) compared to psychological treatments. Table 7 presents the results of logistic regression models comparing the likelihood of chronic course between treatments, and again there was evidence of differences between profile across treatment type received (omnibus test *p* < 0.001). Findings were similar as for relapse; there was evidence that for those in Profile 5, both TAU (OR = 1.98 (95% CI = 1.16; 3.37)) and antidepressants (OR = 2.27 (95% CI = 1.27; 4.06)) were associated with higher odds of a continued chronic compared to psychological treatment. There was also limited evidence that those in Profile 4 were more likely to relapse with treatment as usual compared to psychological therapy (OR = 2.12 (95% CI = 1.00; 4.51)).

Sensitivity analyses conducted with observed (non-imputed) data are presented in Supplementary Online Tables S4 (relapse) and S5 (continued chronic course). Findings for differences for Profile 5 were replicated. Similar to the primary analyses, the odds of relapse were higher when TAU was received compared to psychological treatment for Profile 4 (OR = 2.65 (95% CI = 1.22; 5.78)) and for Profile 6 when antidepressants were received compared to psychological treatments (OR = 2.91 (95% CI = 1.14; 7.42)). These associations were stronger in the observed data compared to those in the primary analyses using imputed data.

## 4. Discussion

This study used latent profile analysis to identify stratified subgroups of patients seeking treatment for depression in primary care. Seven distinct profiles were identified, representing a range of subgroups varying in relation to initial symptom severity, comorbidity, duration of illness, age, employment status, and treatment history. Profiles included a low severity group, to a younger first episode group, an older unemployed group with long duration of illness, and a group with very severe symptoms and longer-term depressive illness. Significant differences in both the likelihood of later relapse in individuals who were previously in remission, and of continued chronic course of illness in those who were not in remission, were observed between profiles. The likelihood of relapse ranged from 17.5% (Profile 1) to 45.7% (Profile 7) between profiles, whilst continued chronic depression ranged from 41.5% (Profile 1) to 77.5% (Profile 7). The profiles could be ranked from highest to lowest likelihood in the same order for each outcome. Psychological treatments were associated with a lower likelihood of relapse and continued chronic course for members of Profile 5 (longer duration of depression and anxiety group), compared to both treatment as usual and antidepressant medications.

### 4.1. Limitations

Whilst this study demonstrates the potential utility of stratification methods in the personalisation of treatment for depression, there are a number of limitations impacting the generalisability of findings. Firstly, this study only included data from seven RCTs, and all were conducted in UK primary care settings, perhaps because the use of the same measure of baseline symptoms and diagnoses (the CIS-R) was an inclusion criterion. The use of the CIS-R was required for data harmonisation purposes, but alternative measures could be used in addition as part of future analyses. The use of RCT data also risks selection biases; for example, there was a lack of ethnic diversity and older mean age of the current sample relative to some other clinical populations with depression in primary care [32]. However, all of the studies were pragmatic trials, increasing the likelihood that the present sample is somewhat representative of other depressed patients presenting in primary care across the world. The fact that all study participants were recruited in primary care also offers an improvement on much of the extant literature, where previously there has often been a lack of information regarding where participants were recruited from [61]. Further, primary care is one of the most common routes into treatment for adults with depression [3,62], so the results here may be generalizable to large proportions of patients with depression. However, the observed profiles may not generalize as well to secondary or inpatient care settings, or to patients seen in different treatment contexts.

The length of follow-up period varied between the studies, and potential changes in depression status between the time points could not be accounted for with the current dataset. This may include further treatments, or complications with antidepressant medication withdrawal, neither of which we could explore in the current dataset. More frequent measurement of depression symptoms following the end of treatment could allow for the identification of the clinical profiles of patients who are likely to relapse earlier, which would further their utility in supporting clinical decision making.

Although data came from participants of RCTs, we analysed data post-treatment and across studies, losing the benefits of randomization in controlling for confounding. Furthermore, a number of potential confounding factors were not available in the dataset, and some of these factors have also been associated with increased risk of relapse. For example, childhood maltreatment, neuroticism, rumination, and interpersonal stress have all been linked to increased risk of relapse [6]. As well as using these factors in future analyses, the utility of profiling approaches to identify at-risk groups may also be enhanced with subgroup specific factors not available here. For example, although gender is not independently prognostic for depression treatment outcomes or course [6,63], and does not typically discriminate between latent profiles of patients with depression [39,40], data on pregnancy or menopause may have helped further refine the latent profiles identified in this study [64,65,66]. In addition, none of the randomised treatments included relapse prevention specific psychological treatments (e.g., mindfulness based cognitive therapy or continuation CBT) [29], a number of common antidepressant medications were not represented in the studies included here, and none included starting combination therapies or augmentation strategies for those not in remission at the primary end-point, so consideration of differential benefits between types of treatment were somewhat limited here. It is also noteworthy that some participants randomized to treatment as usual at baseline may have received a course of antidepressants during the trial. A further limitation to the analysis was due to the focus on continued chronic course of depression and relapse as outcomes. The relapse outcome required participants to have been in remission 3 to 4 months after starting treatment, and therefore individuals who did not remit (over 60% of the sample) were excluded for analyses of this outcome. Whilst unavoidable, this loss of sample will have impacted the available power for analyses. Linked to this, despite the inclusion of seven RCTs, the final sample sizes for comparisons between profiles were small for some estimates, and especially when further grouping by treatment received, limiting the available power to detect differences within profiles. This highlights the need for sufficient sample sizes to explore the further utility of stratification methods to inform clinical practice for adults with depression, and the actual clinical utility of these profiles can only be assessed through a prospective study. Further, there were multiple comparisons made, and it might be argued that adjustments could have been performed to account for the likelihood of Type 1 errors. However, following Rothman [67], and because all analyses followed a pre-registered analysis plan, this was not deemed necessary for this exploratory analysis. Finally, the use of these outcomes might have introduced additional bias in the assessment of differences within profiles across treatment types. If it were the case that one treatment led to a much greater chance of being in remission at 3 to 4 months than another, comparing outcomes after this time-point would have led to considerable selection bias. However, as the results were consistent across the two primary outcomes, this type of selection bias seems to be unlikely in the present study.

### 4.2. Implications

Findings from this study highlight the potential use of patient stratification methods to identify subgroups of individuals suffering from depression who are more or less likely to follow different courses of illness. Some of these individuals, such as those in Profiles 6 and 7, were at particularly high risk of continued chronic illness, or of later relapse. Conversely, patients in Profiles 1 and 3 were at relatively lower risk of continued chronic depression or relapse. The identification of these groups, potentially at the point of pre-treatment assessment, or a first consultation with a clinician might help inform treatment planning to mitigate these long-term risks. This might involve consideration of more or less regular reviews with a clinician, more aggressive treatments or the augmentation of treatments to prevent chronic depression [30,68], or more conservative management and the use of relapse prevention focussed psychological therapies. Relapse prevention focussed cognitive behavioural therapy, mindfulness based cognitive therapy, or interpersonal psychotherapy have all proven efficacious in reducing the risk of relapse up to one year post-treatment [29]. Information on the risks of chronic depression and relapse might also inform treatment decisions on continuation or discontinuation of medications [69,70]. Additionally, the present study has also provided an analysis which, if replicated in a larger sample, might suggest that psychological therapies could be more beneficial for mitigating the risks of both relapse and continued chronic depression for individuals in Profile 5, relative to treatment as usual or treatment with antidepressant medications. Members of this profile were highly likely to have been suffering from both depression and anxiety for more than one year, and to have a history of antidepressant use, and made up 20% of the analytic sample. Further, in sensitivity analyses, there appeared to be a benefit of psychological therapies compared to treatment as usual and antidepressants for those in Profiles 4 and 6, respectively. If these findings were replicated on external data, it might support calls to increase access to psychological therapies [32] and could potentially inform personalization of care [71]. The risks of both continued chronic depression and relapse were quite high across this study sample. Given the limitations noted above, to better understand how these phenomena can be mitigated and the ways in which treatment can be optimally personalized to do so, it is particularly important that treatment studies continue to collect data to capture these common outcomes, and in routine care services, follow-up appointments and relapse prevention interventions are normalized and integrated into usual practices [32,72].

### 4.3. Conclusions

The potential utility of patient stratification approaches for supporting clinical decision making have been demonstrated in areas of physical healthcare, and to a lesser extent in predicting response in mental health treatments, but research has not considered the value of stratification for predicting long-term outcomes. This novel study presents a patient stratification approach that identified subgroups of participants who were either more or less likely to either relapse or follow a continued chronic course of depressive illness following treatment for depression. Seven profiles were identified using baseline severity, duration of illness, history of antidepressant use, employment status, and age. Profiles included young people presenting with their first episode, older people with more chronic illness, and individuals with severe symptoms and chronic illness pre-treatment. Substantial differences in the likelihood of relapse and following a continued chronic course between profiles were observed, with some profiles at particular risk of both later relapse and of chronic course of illness. Treatment type was not consistently associated with different outcomes in most profiles, perhaps due to the numbers of participants in stratified profile-by-treatment-groups being low overall, making it difficult to confidently ascertain differences. However, members of Profile 5 appeared to have better outcomes with psychological therapies compared to either antidepressants or treatment as usual, and there was some evidence that members of Profiles 4 and 6 may also potentially have had better outcomes from psychological therapy. This study demonstrates the potential utility of patient identification approaches for ascertaining the likelihood of different long-term outcomes, thereby supporting clinical decision making. The findings might inform subgroups of patients for whom the provision of addition treatments are likely to be needed due to poorer initial response, as well as interventions to reduce the risk of relapse for those who do initially respond.

## Figures and Tables

**Table 1:** Description of included studies from the Dep-GP IPD dataset.

Study	N	Sample	Interventions	Primary Outcome Measure	Follow Up Time Points (Months from Baseline)
CADET	581	Adults ≥18, ICD-10 depressive episode	Collaborative Care + TAU vs. TAU	PHQ-9	4 and 12
COBALT	469	Adults 18–75 with treatment resistant depression, scoring ≥14 BDI-II	TAU vs. CBT + TAU	PHQ-9 (& BDI-II at baseline)	3, 6, and 9
IPCRESS	299	Adults scoring ≥14 BDI-II and GP confirmed diagnosis of depression	iCBT vs. TAU	BDI-II	4 and 6
MIR	480	Adults ≥18 taking SSRIs or SNRIs at adequate dose for ≥6 weeks, and scored ≥14 on BDI-II	SSRIs or SNRIs + Mirtazapine vs. SSRIs or SNRIs + Pill Placebo	BDI-II (also PHQ-9)	3, 6, and 12
REEACT	691	Adults with PHQ-9 > 10 presenting to GP with depression	Moodgym vs. Beating the Blues vs. TAU	PHQ-9	4 and 12
RESPOND	220	Women meeting criteria for MDD within 6-months post-partum	ADM vs. listening intervention	EPDS	4 and 10
TREAD	361	Adults 18–69 who met diagnostic criteria for MDD and scored ≥14 on BDI-II	TAU vs. Physical Activity + TAU	BDI-II	4, 7, and 12

Notes: MDD: Major depressive disorder; TAU: Treatment as usual; iCBT: internet-delivered CBT; ADM: Antidepressant medication.

**Table 2:** Descriptive statistics of the sample.

Continuous Variables	N	M (sd)
Depression severity score	3037	13.8 (3.4)
Anxiety severity score	3037	14.0 (6.6)
Age	3037	42.6 (13.8)
**Categorical Variables**	**Category**	**N (%)**
Employed (N (%))	Yes	1511 (49.8%)
	No	1524 (50.2%)
	Missing	2 (0.1%)
Duration of depression	Less than 2 weeks	42 (1.4%)
	2 weeks to 6 months	937 (30.9%)
	6 to 12 months	564 (18.6%)
	1 and 2 years	457 (15.1%)
	More than 2 years	1037 (34.2%)
Duration of anxiety	Less than 2 weeks	6 (0.2%)
	2 weeks to 6 months	342 (11.3%)
	6 to 12 months	299 (9.9%)
	1 and 2 years	320 (10.5%)
	More than 2 years	1385 (45.6%)
	Missing	685 (22.6%)
History of antidepressant use	Yes	2069 (68.1%)
	No	965 (31.8%)
	Missing	3 (0.1%)
Sex	Male	868 (28.6%)
	Female	2167 (71.4%)
	Missing	2 (0.1%)
Ethnicity group	White	2840 (93.5%)
	Black and Minority Ethnic	195 (6.4)
	Missing	2 (0.1%)
Treatment type	Treatment as usual	1076 (35.4%)
	Psychological interventions	1079 (35.5%)
	Antidepressant medication	589 (19.4%)
	Other	293 (9.7%)

**Table 3:** Descriptive statistics for baseline variables in each identified profile.

		Profile 1 (5%)	Profile 2 (10%)	Profile 3 (15%)	Profile 4 (19%)	Profile 5 (20%)	Profile 6 (10%)	Profile 7 (22%)
Depression severity *		6.1 (2.0)	12.4 (3.3)	11.8 (2.1)	16.0 (1.9)	13.0 (2.3)	12.2 (2.4)	16.8 (2.0)
Anxiety severity *		6.2 (3.7)	10.3 (4.1)	9. 8 (4.2)	18.4 (4.7)	11.1 (4.4)	10.1 (4.5)	20.8 (4.9)
Age *		41.8 (13.0)	29.1 (8.5)	40.6 (10.8)	37.7 (11.6)	41.6 (11.4)	63.3 (7.2)	46.3 (12.2)
Depression 1+ year ^§^	Yes	48 (34.5%)	5 (1.6%)	8 (1.8%)	15 (2.6%)	607 (99.7%)	187 (62.1%)	624 (94.8%)
	No	91 (65.5%)	311 (98.4%)	433 (98.2%)	558 (97.4%)	2 (0.3%)	114 (37.9%)	34 (5.2%)
Anxiety + 1 year ^§^	Yes	26 (18.7%)	65 (20.6%)	117 (26.5%)	216 (37.7%)	467 (76.7%)	227 (75.4%)	587 (89.2%)
	No	12 (8.6%)	184 (58.2%)	154 (34.9%)	262 (45.7%)	4 (0.7%)	26 (8.6%)	0
	Missing	101 (72.7%)	67 (21.2%)	170 (38.6%)	95 (16.6%)	138 (22.7%)	48 (15.6%)	71 (10.8%)
History ADM use ^§^	Yes	73 (52.5%)	144 (45.6%)	256 (58.1%)	398 (69.5%)	455 (74.7%)	233 (77.4%)	510 (77.5%)
	No	65 (46.8%)	172 (54.4%)	184 (41.7%)	174 (30.4%)	154 (25.3%)	68 (22.6%)	148 (22.5%)
	Missing	1 (0.7%)	0	1 (0.2%)	1 (0.2%)	0	0	0
Employed ^§^	Yes	98 (70.5%)	0	441 (100%)	329 (57.4%)	430 (70.6%)	0	213 (32.4%)
	No	41 (29.5%)	316 (100%)	0	243 (42.4%)	178 (29.2%)	301 (100%)	445 (67.6%)
	Missing	0	0	0	1 (0.2%)	1 (0.2%)	0	0

Note: * Means and standard deviations presented; ^§^ N and % presented; ADM: antidepressant medication.

**Table 4:** Associations with relapse for each profile compared to two reference groups: the largest overall profile (Profile 7) and the profile with approximately equal numbers in remission and not in remission at 3 to 4 months (Profile 3).

	Reference = Profile 7			Reference = Profile 3	
	OR	95% CI		OR	95% CI
Profile 1	0.30	(0.13; 0.68)	Profile 1	0.79	(0.37; 1.70)
Profile 2	0.51	(0.27; 0.97)	Profile 2	1.35	(0.73; 2.50)
Profile 3	0.38	(0.22; 0.65)	Profile 3	Ref	Ref
Profile 4	0.69	(0.41; 1.16)	Profile 4	1.82	(1.11; 2.99)
Profile 5	0.44	(0.27; 0.73)	Profile 5	1.16	(0.70; 1.93)
Profile 6	0.68	(0.40; 1.15)	Profile 6	1.78	(1.04; 3.04)
Profile 7	Ref	Ref	Profile 7	2.63	(1.53; 5.43)

Note: Odds ratios and confidence intervals are adjusted for the randomised treatment in each RCT.

**Table 5:** Associations with continued chronic course of depression for each profile compared to two reference groups: the largest overall profile (Profile 7) and the profile with approximately equal numbers in remission and not in remission at 3 to 4 months (Profile 3).

	Reference = Profile 7			Reference = Profile 3	
	OR	95% CI		OR	95% CI
Profile 1	0.23	(0.12; 0.46)	Profile 1	0.74	(0.67; 1.47)
Profile 2	0.41	(0.25; 0.68)	Profile 2	1.29	(0.75; 2.19)
Profile 3	0.32	(0.21; 0.47)	Profile 3	Ref.	Ref.
Profile 4	0.58	(0.41; 0.83)	Profile 4	1.84	(1.24; 2.72)
Profile 5	0.39	(0.28; 0.54)	Profile 5	1.24	(0.83; 1.83)
Profile 6	0.56	(0.38; 0.84)	Profile 6	1.77	(1.11; 2.82)
Profile 7	Ref.	Ref.	Profile 7	3.15	(2.11; 4.71)

Note: Odds ratios and confidence intervals are adjusted for the randomised treatment in each RCT.

**Table 6:** Association between treatment types and relapse within each profile.

	TAU (vs. Psychological Interventions)		Antidepressants (vs. Psychological Interventions)		Antidepressants (vs. TAU)	
	OR	95% CI	OR	95% CI	OR	95% CI
Profile 1 (*n* = 87)	2.18	(0.52; 9.11)	4.31	(0.57; 32.84)	1.98	(0.29; 13.68)
Profile 2 (*n* = 118)	2.39	(0.76; 7.54)	1.02	(0.29; 3.58)	0.42	(0.13; 1.37)
Profile 3 (*n* = 207)	1.15	(0.53; 2.46)	1.23	(0.31; 4.94)	1.07	(0.26; 4.38)
Profile 4 (*n* = 178)	2.12	(1.00; 4.51)	0.55	(0.14; 2.20)	0.26	(0.06; 1.07)
Profile 5 (*n* = 215)	2.51	(1.16; 5.40)	2.92	(1.24; 6.87)	1.16	(0.52; 2.59)
Profile 6 (*n* = 128)	1.92	(0.76; 4.83)	2.40	(0.96; 5.96)	1.25	(0.50; 3.12)
Profile 7 (*n* = 120)	1.36	(0.59; 3.16)	1.91	(0.70; 5.21)	1.40	(0.53; 3.70)

Note: Treatment in parentheses is the reference category.

**Table 7:** Association between treatment type and a continued chronic course of depression within each profile.

	TAU (vs. Psychological Interventions)		Antidepressants (vs. Psychological Interventions)		Antidepressants (vs. TAU)	
	OR	95% CI	OR	95% CI	OR	95% CI
Profile 1 (*n* = 50)	2.35	(0.57; 9.61)	2.51	(0.45; 14.07)	1.07	(0.19; 5.95)
Profile 2 (*n* = 115)	1.12	(0.41; 3.04)	0.61	(0.16; 2.30)	0.54	(0.16; 1.89)
Profile 3 (*n* = 187)	1.70	(0.81; 3.54)	0.86	(0.32; 2.29)	0.50	(0.19; 1.34)
Profile 4 (*n* = 303)	0.93	(0.50; 1.72)	1.21	(0.49; 2.98)	1.30	(0.55; 3.04)
Profile 5 (*n* = 365)	1.98	(1.16; 3.37)	2.27	(1.27; 4.06)	1.15	(0.64; 2.06)
Profile 6 (*n* = 162)	1.18	(0.52; 2.68)	1.94	(0.84; 4.47)	1.64	(0.73; 3.70)
Profile 7 (*n* = 509)	1.22	(0.75; 2.00)	1.60	(0.84; 3.03)	1.31	(0.70; 2.43)

Note: Treatment listed in parentheses is the reference category.

## Data Availability

Requests for sharing of the IPD used in this study can be made to the corresponding author, any sharing of data will be subject to obtaining appropriate agreements from the chief investigators or data custodians for each individual trial dataset used here.

## Supplementary Materials

The following are available online at https://www.mdpi.com/article/10.3390/jpm11121295/s1, Table S1: Model fit statistics, Table S2: Associations with relapse for each profile compared to two reference groups, the largest overall profile (profile 7) and the profile with approximately equal numbers in remission and not in remission at 3-to-4 months (profile 3) (observed data), Table S3: Associations with continued chronic course of depression for each profile compared two reference groups, the largest overall profile (profile 7) and the profile with approximately equal numbers in remission and not in remission at 3-to-4 months (profile 3) (observed data), Table S4: Association between treatment types and relapse within each profile (observed data), Table S5: Association between treatment type and continued chronic depression within each profile (observed data), Table S6: Ethical approval and Trial Registration details of the included studies from the Dep-GP IPD database.
