# Supplementary material for: A Patient Stratification Approach to Identifying the Likelihood of Continued Chronic Depression and Relapse Following Treatment for Depression
Source: J Pers Med. 2021 Dec 4;11(12):1295. doi: 10.3390/jpm11121295 (PMC8703621; doi:10.3390/jpm11121295)
Supplement: Supplementary file 1 [file jpm-11-01295-s001.zip › jpm-1405561-SI.pdf]

Supplementary online materials to:

A patient stratification approach to identifying the likelihood of continued chronic depression and relapse following treatment for depression

Content:

Supplementary Materials A: Model fit statistics for latent profile analysis. (Page 2)

Supplementary Materials B: Observed case analysis: Between profile comparisons. (Page 3)

Supplementary Materials C: Observed case analysis: Between treatment comparisons. (Page 4)

Supplementary Materials D: Ethical Approvals for the included studies (Page 5)

Supplementary Materials A: Model fit statistics for latent profile analysis.

Table S1: Model fit statistics.

Profile Solution	AIC	BIC	SABIC	VLMR-LRT p-value	Entropy	Classification (% per profile)
2-Class	74265	74373	74316	0.000	0.689	44/55
3-Class	73744	73900	73818	0.001	0.668	30/27/43
4-Class	73377	73582	73474	0.000	0.667	28/31/15/26
5-Class	73233	73486	73352	0.028	0.672	7/19/33/19/21
6-Class	73100	73401	73242	0.001	0.671	7/26/18/9/22/18
**7-Class**	**73031**	**73380**	**73196**	**0.019**	**0.716**	**5/10/15/19/20/10/22**
8-Class	72924	73322	73112	0.434	0.690	7/12/17/10/12/15/16/12

Supplementary Materials B: Observed case analysis: Between profile comparisons.

Table S2. Associations with relapse for each profile compared to two reference groups, the largest overall profile (profile 7) and the profile with approximately equal numbers in remission and not in remission at 3-to-4 months (profile 3) (observed data).

	Reference = Profile 7)			Reference = Profile 3)	
	OR	95% CI		OR	95% CI
Profile 1	0.34	(0.15;0.76)	Profile 1	0.89	(0.41;1.95)
Profile 2	0.62	(0.32;1.19)	Profile 2	1.63	(0.86;3.11)
Profile 3	0.38	(0.22;0.66)	Profile 3	Ref	Ref
Profile 4	0.72	(0.42;1.24)	Profile 4	1.92	(1.12;3.28)
Profile 5	0.46	(0.28;0.77)	Profile 5	1.23	(0.73;2.06)
Profile 6	0.75	(0.44;1.28)	Profile 6	1.98	(1.13;3.45)
Profile 7	Ref	Ref	Profile 7	2.65	(1.52;4.63)

Note: Odds ratios and confidence intervals are adjusted for the randomised treatment in each RCT

Table S3. Associations with continued chronic course of depression for each profile compared two reference groups, the largest overall profile (profile 7) and the profile with approximately equal numbers in remission and not in remission at 3-to-4 months (profile 3) (observed data).

	Reference = Profile 7)			Reference = Profile 3)	
	OR	95% CI		OR	95% CI
Profile 1	0.21	(0.10;0.41)	Profile 1	0.83	(0.40;1.71)
Profile 2	0.34	(0.20;0.57)	Profile 2	1.37	(0.78;2.39)
Profile 3	0.25	(0.16;0.38)	Profile 3	Ref	Ref
Profile 4	0.49	(0.34;0.72)	Profile 4	2.00	(1.30;3.07)
Profile 5	0.34	(0.25;0.48)	Profile 5	1.38	(0.92;2.09)
Profile 6	0.51	(0.34;0.77)	Profile 6	2.05	(1.26;3.34)
Profile 7	Ref	Ref	Profile 7	4.04	(2.65;6.17)

Note: Odds ratios and confidence intervals are adjusted for the randomised treatment in each RCT

Supplementary Materials C: Observed case analysis: Between treatment comparisons.

The tables below present sensitivity analyses for differences in the likelihood of relapse and continued chronic course of illness in the sub-sample of participants with complete data (i.e. no imputation was required). For some comparisons involving Profiles 1, 2,3 and 4, there were less than 20 participants receiving at least one of the treatment types and therefore these estimates were not considered sufficiently robust to be presented, they are instead represented with an asterix "*" in the tables below.

Table S4. Association between treatment types and relapse within each profile (observed data)

	TAU (vs Psychological interventions)		Antidepressants (vs Psychological interventions)		Antidepressants (vs TAU)	
	OR	95% CI	OR	95% CI	OR	95% CI
Profile 1 (n=63)	2.76	(0.62;12.32)	*	*	*	*
Profile 2 (n=89)	2.28	(0.71;7.37)	0.72	(0.18;2.87)	0.32	(0.09;1.17)
Profile 3 (n=155)	1.24	(0.55;2.80)	*	*	*	*
Profile 4 (n=139)	2.65	(1.22;5.78)	*	*	*	*
Profile 5 (n=186)	2.79	(1.29;6.05)	2.84	(1.19;6.78)	1.02	(0.44;2.35)
Profile 6 (n=117)	1.88	(0.74;4.76)	2.91	(1.14;7.42)	1.55	(0.61;3.95)
Profile 7 (n=110)	1.34	(0.57;3.15)	1.74	(0.63;4.78)	1.29	(0.48;3.51)

Note: Treatment in parentheses is the reference category.

Table S5. Association between treatment type and continued chronic depression within each profile (observed data)

	TAU (vs Psychological interventions)		Antidepressants (vs Psychological interventions)		Antidepressants (vs TAU)	
	OR	95% CI	OR	95% CI	OR	95% CI
Profile 1 (n=39)	*	*	*	*	*	*
Profile 2 (n=70)	0.72	(0.24;2.17)	*	*	*	*
Profile 3 (n=134)	2.00	(0.94;4.27)	0.92	(0.34;2.51)	0.46	(0.17;1.23)
Profile 4 (n=200)	0.90	(0.46;1.74)	1.37	(0.53;3.55)	1.52	(0.62;3.75)
Profile 5 (n=294)	2.30	(1.31;4.02)	2.77	(1.54;5.01)	1.21	(0.66;2.20)
Profile 6 (n=150)	1.23	(0.53;2.85)	1.98	(0.84;4.67)	1.61	(0.71;3.65)
Profile 7 (n=421)	1.26	(0.76;2.09)	1.89	(0.98;3.65)	1.50	(0.78;2.90)

Note: Treatment in parentheses is the reference category.

Supplementary Materials D: Ethical Approvals for the included studies

Ethical Standards

The authors assert that all procedures contributing to this work comply with the ethical standards of the relevant national and institutional committees on human experimentation and with the Helsinki Declaration of 1975, as revised in 2008. The authors assert that all procedures contributing to this work comply with the ethical standards of the relevant national and institutional guides on the care and use of laboratory animals.

Table S6. Ethical approval and Trial Registration details of the included studies from the Dep-GP IPD database.

Study	Ethical Approvals	Trial Registration details
CADET	Granted by NHS Health Research Authority & NRES Committee South West (NRES/07/H1208/60)	ISRCTN32829227; https://doi.org/10.1186/ISRCTN32829227
COBALT	Approvals were granted by West Midlands Research Ethics Committee (NRES/07/H1208/60) and research governance approval was obtained from the local Primary Care Trusts/Health Boards	ISRCTN38231611; https://doi.org/10.1186/ISRCTN38231611
IPCRESS	Approval granted by Royal Free and Hampstead Research Ethics Committee, reference number 05/Q0501/18	ISRCTN45444578; https://doi.org/10.1186/ISRCTN45444578
MIR	Approvals were granted by South East Wales Research Ethics Committee Panel C (ref: 12/WA/0353); Bristol Clinical Commissioning Group (CCG), and other CCGs provided research governance assurance.	ISRCTN06653773; https://doi.org/10.1186/ISRCTN06653773
REEACT	The Leeds (East) research ethics committee granted approval (08/H1306/77).	ISRCTN91947481; https://doi.org/10.1186/ISRCTN91947481
RESPOND	Approvals were granted by the Scotland A Multi-centre Research Ethics Committee (MREC; reference number MREC/03/0/127) and site-specific approval was obtained from 10 relevant local ethics committees and 10 primary care trusts (PCTs)	ISRCTN16479417; https://doi.org/10.1186/ISRCTN16479417
TREAD	Approvals were granted by West Midlands multicentre research ethics committee (MREC 05/MRE07/42), and research governance approval was given by the relevant local National Health Service primary care trusts	ISRCTN16900744; https://doi.org/10.1186/ISRCTN16900744
